# Triple-negative breast cancer targeting and killing by EpCAM-directed, plasmonically active nanodrug systems

**DOI:** 10.1038/s41698-017-0030-1

**Published:** 2017-09-01

**Authors:** Samir V. Jenkins, Zeid A. Nima, Kieng B. Vang, Ganesh Kannarpady, Dmitry A. Nedosekin, Vladimir P. Zharov, Robert J. Griffin, Alexandru S. Biris, Ruud P. M. Dings

**Affiliations:** 1 Department of Radiation Oncology, University of Arkansas for Medical Sciences, Little Rock, AR USA; 2 Center for Integrative Nanotechnology Sciences, University of Arkansas at Little Rock, Little Rock, AR USA; 3 The Phillips Classic Laser and Nanomedicine Laboratories, University of Arkansas for Medical Sciences, Little Rock, AR USA

## Abstract

An ongoing need for new cancer therapeutics exists, especially ones that specifically home and target triple-negative breast cancer. Because triple-negative breast cancer express low or are devoid of estrogen, progesterone, or Her2/Neu receptors, another target must be used for advanced drug delivery strategies. Here, we engineered a nanodrug delivery system consisting of silver-coated gold nanorods (AuNR/Ag) targeting epithelial cell adhesion/activating molecule (EpCAM) and loaded with doxorubicin. This nanodrug system, AuNR/Ag/Dox-EpCAM, was found to specifically target EpCAM-expressing tumors compared to low EpCAM-expressing tumors. Namely, the nanodrug had an effective dose (ED_50_) of 3 μM in inhibiting 4T1 cell viability and an ED_50_ of 110 μM for MDA-MD-231 cells. Flow cytometry data indicated that 4T1 cells, on average, express two orders of magnitude more EpCAM than MDA-MD-231 cells, which correlates with our ED_50_ findings. Moreover, due to the silver coating, the AuNR/Ag can be detected simultaneously by surface-enhanced Raman spectroscopy and photoacoustic microscopy. Analysis by these imaging detection techniques as well as by inductively coupled plasma mass spectrometry showed that the targeted nanodrug system was taken up by EpCAM-expressing cells and tumors at significantly higher rates than untargeted nanoparticles (*p* < 0.05). Thus, this approach establishes a plasmonically active nanodrug theranostic for triple-negative breast cancer and, potentially, a delivery platform with improved multimodal imaging capability for other clinically relevant chemotherapeutics with dose-limiting toxicities, such as platinum-based or taxane-based therapies.

## Targeted drug delivery for triple-negative breast cancer

Silver-coated gold nanorods deliver drugs to a difficult-to-treat breast cancer by targeting an over-expressed antigen on its surface. Ruud Dings and colleagues at the University of Arkansas in the USA loaded the chemotherapeutic drug doxorubicin onto silver-coated gold nanorods that were conjugated with an antibody that specifically targets an over-expressed antigen on many types of ‘triple-negative breast cancers’ (TNBCs). Unlike other breast cancers, TNBCs lack certain receptors, making them difficult to target with cancer therapies. The team found that one of the two TNBC cell lines studied over-expressed the epithelial antigen EpCAM 100 times more than the other. Their drug-loaded silver-coated gold nanorods specifically targeted the EpCAM over-expressing cells over the low-expressing ones. The nanorods’ coatings also allowed them to be easily detected by two different imaging techniques: surfaced-enhanced Raman spectroscopy and photoacoustic microscopy.

## Introduction

The number of new anti-cancer agents approved by the US Food and Drug Administration (FDA) has decreased by more than 50% over the past decade, and most of the newly approved drugs target molecular entities via the same pathway(s) as previously approved therapeutics.^1, 2^ Clinically, these compounds are prescribed at their maximal tolerated dose due to their toxicity, i.e., unwanted off-target side effects.^3^ As a result, the drug concentrations achieved within the local tumor microenvironment are limited and likely to be below the most effective dose. Drugs are further hampered by the rapid emergence of therapy resistance in the body, consequently hindering long-term clinical success. Anthracyclines such as doxorubicin (Dox) are first-line, key components in many anti-cancer treatment strategies, including breast and ovarian cancer, but toxicity remains an important concern.^4, 5^ These factors have created a strong, ongoing need to enhance the specificity and effectiveness of therapeutics that are already in clinical use.

One attempt to meet this need was the development of liposome-encapsulated doxorubicin (Doxil).^6, 7^ Doxil relies on the enhanced permeability and retention effect to passively accumulate in the tumor tissue.^6, 7^ Polymeric and dendrimer nanoparticles are also in development as alternative delivery vehicles. Similarly, a number of gold-based nanomaterials are being investigated to deliver other challenged chemotherapeutics to tumors.^8–10^ The biocompatibility and tunable surface chemistry of these materials enables conjugation of drug moieties to the surface. These molecules can then be released under the unique environmental conditions of a tumor, e.g., a lower pH, or in response to an external stimulus, in particular laser irradiation. In general, a significant drawback of both Doxil and many other nanoparticle-based therapeutics under development is their lack of targeting specificity, i.e., a targeting moiety, which would enhance delivery to the tumor tissue.

Efficient targeting of tumors would also allow reduced doses of chemotherapeutics to be used, thus reducing the likelihood of unwanted toxicity. Triple-negative breast cancer (TNBC) is particularly difficult to target because it does not or at low levels at best express estrogen, progesterone, or Her2/Neu receptors—signaling pathways involved at least in part for many current FDA-approved breast cancer therapeutics. The epithelial cell adhesion/activating molecule (EpCAM; CD326) is a transmembrane surface epithelial differentiation antigen that mediates epithelium-specific Ca^2+^-independent homotypic cell–cell adhesions. It is expressed on the surface of many TNBCs, making it an attractive target for this malignancy.^11, 12^ TNBC is a heterogeneous group and tumors with EpCAM overexpression are correlated with poor disease-free and poor overall survival.^12^ High expression of EpCAM is detected in ~45% of aggressive invasive ductal breast carcinomas cases, whereas invasive lobular breast carcinomas only show high EpCAM expression in 15% of cases.^13, 14^ While EpCAM was one of the earliest cancer-selective targets identified, drugs targeting it alone have not made significant progress in the clinic. In addition, the use of EpCAM targeting by multifunctional nanotherapeutics has not been extensively explored. The overexpression of EpCAM by TNBC makes it a viable target for next-generation nanoparticle therapeutics.

In this work, we covalently loaded Dox onto poly(ethylene glycol)-stabilized (PEGylated), silver-coated gold nanorods (AuNR/Ag), then conjugated them with an EpCAM antibody (anti-EpCAM) to treat and target TNBC. Conjugation with *p*-aminothiophenol (PATP) provides a unique, trackable Raman signature, and the high optical absorption by AuNRs generates a strong photoacoustic (PA) signal. These complementary methods allow the AuNRs to be readily distinguished from the complex biological background. In our previous work, we have used these unique signatures to accurately detect and identify circulating tumor cells among millions of blood cells.^15, 16^ The Ag layer (1–2 nm) was shown to significantly (around 200 times) increase the intensity of the surface-enhanced Raman spectroscopy (SERS) signature, facilitating SERS-based detection.^15, 16^ Therefore, the combination of SERS and PA, as well as precise targeted drug delivery could result in an extremely effective multimodal theranostic platform for detection and treatment of various cancers.

Here, we show expression levels of EpCAM by conventional flow cytometry and the ability of our targeted AuNRs to specifically interact with EpCAM-expressing cells via PA flow cytometry. Treatment of EpCAM-expressing cells resulted in a nearly 10-fold lower effective dose (ED_50_) than when untargeted drug-loaded AuNRs were administered. Moreover, we were able to detect nanoparticle-tumor cell association in vitro and in vivo using Raman and PA mapping as well as by inductively coupled plasma mass spectrometry (ICP-MS). The retention and uptake profiles suggest our nanomaterial-based imaging and delivery strategy warrant further studies in pre-clinical in vivo models.

## Results

### Particle synthesis and characterization

**Fig. 1:**
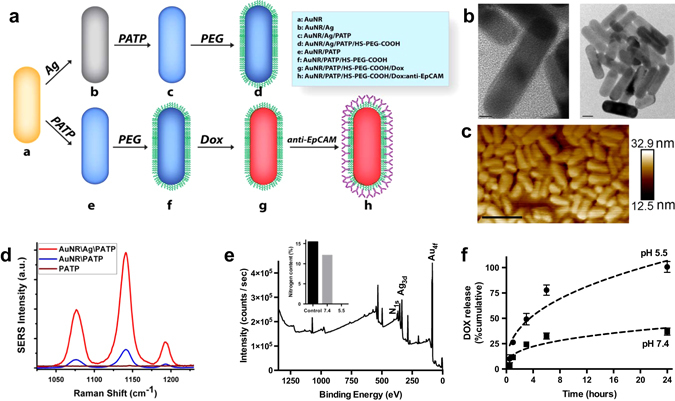
Synthesis and characterization of gold nanorods. **a** Schematic illustration of synthesis of EpCAM-targeting AuNR/Ag loaded with doxorubicin. Created by Z.A. Nima. **b** Transmission electron microscopy (*TEM*) of AuNR formulations, showing an average length of 36 nm and width of 12 nm, an aspect ratio of 3.* Scale bar* in the* left panel* represents 5 nm, *right panel* 10 nm. **c** Representative image of an atomic force microscopy (*AFM*) scan of the AuNR/Ag. *Scale bar* represents 100 nm. **d** Raman signal enhancement of Ag seen at 1080 cm^-1^. **e** Representative X-ray photoelectron spectroscopy (*XPS*) wide scan for AuNR/Ag/Dox. **f** Dynamics and kinetics of Dox release over 24 h at pH 7.4 and pH 5.5

AuNR/Ags were synthesized with an average length of ~36 nm and an average width of ~12 nm (aspect ratio of around 3), based on transmission electron microscopy and atomic force microscopy (AFM) measurements (Fig. 1). The transverse and longitudinal localized surface plasmon resonance peaks appeared at 520 nm and 740 nm, respectively, whereas the integrated SERS signal was measured at 1080 cm^-1^. Addition of the Ag layer led to ~200 fold enhancement of this signal (Fig. 1d). The additions of Dox or EpCAM antibody did change the Raman spectrum significantly (Supplementary Fig. 1).

Subsequently, Dox was conjugated to PEG via 1-ethyl-3-(3-(dimethylamino)- propyl) carbodiimide ﻿(EDC)/N﻿-hydroxysuccinimide (NHS) coupling between the carboxyl terminus of PEG and the primary amine in Dox, which yielded a bulk average 4% (w/w) loading of Dox (based on UV–Vis analysis). A 100 µg/ml solution of AuNR/Ag corresponds to roughly 7 µM Dox. Next, anti-EpCAM antibodies were conjugated to available PEG termini on both non-loaded and Dox-loaded constructs.

### Doxorubicin release dynamics and kinetics

The kinetics of Dox release was assessed by X-ray photoelectron spectroscopy (XPS) and UV–Vis spectroscopy at different pHs, namely physiological pH (7.4) and a more acidic pH (5.5) found in and around hypoxic regions of the tumor (Fig. 1e, f). Using XPS the atomic concentration of various elements was calculated using the area under the peaks for respective elements. The normalized nitrogen atomic concentration, representative of Dox bound to the surface of the particle, was 15.5% (Fig. 1e, *inset*). This dropped to 12.1% (22% decrease or release) after 24 h exposure to physiological pH 7.4, while it was undetectable after 24 h at pH 5.5. This observation was corroborated by UV–Vis spectroscopy (Fig. 1f), which showed that after 24 h around 25% of Dox was released at pH 7.4, yet at the pH of 5.5, 50% of the Dox was released after 4 h and 100% after 24 h.

### EpCAM expression profiles of breast cancer cell lines

**Fig. 2:**
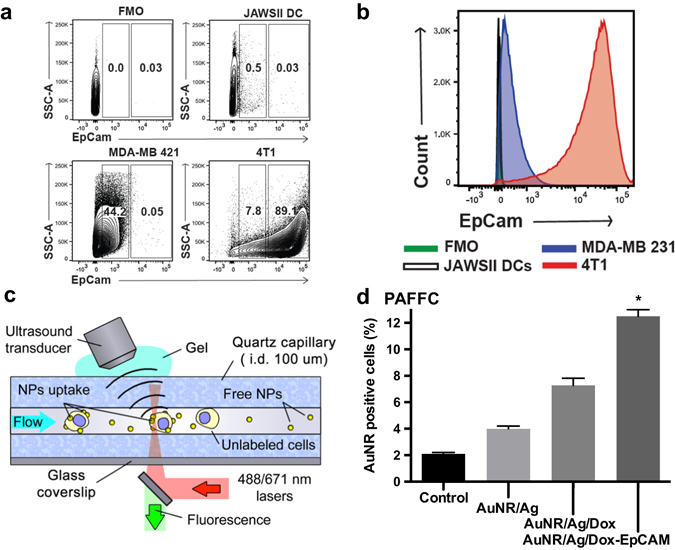
A 100-fold differential of EpCAM expression on TNBC cells. **a** Representative dot plots of EpCAM expression on 4T1 and MDA-MB-231 breast cancer cells and JAWSII, normal non-cancerous dendritic cells. **b** 4T1 TNBC cells express 100-fold more EpCAM than MDA-MB-231 cells and 1000-fold more than JAWSII dendritic cells. The fluorescence minus one is included as a control. **c** Visual representation of integrated PA and fluorescent flow cytometry (*PAFFC*). Created by D.A. Nedosekin. **d** Quantification of AuNR/Ag/Dox-EpCAM photoacoustic signals from intact fluorescein diacetate stained 4T1 cells by PAFFC. Data represent mean ± SEM

EpCAM expression was markedly greater in 4T1 cells than in MDA-MB-231 cells. We detected a near 100-fold difference in EpCAM expression in 4T1 and MDA-MB-231 cells (Fig. 2a, b). JAWSII dendritic cells, a non-breast cancer cell line, as well as the fluorescence minus one controls were negative. Specifically, EpCAM expression was divided into two gates based on high or low expression of the EpCAM receptor (Fig. 2a). Based on the high expression population, EpCAM was expressed by 89.1% of 4T1 cells compared to 0.05% of MDA-MB-231, while the low expression EpCAM population was seen in only 7.8% of 4T1 cells, in contrast to 44.2% of MDA-MB-231 cells.

Subsequently, we used PAFFC to quantify AuNR/Ag association (Fig. 2c). We found that the AuNR/Ag/Dox-EpCAM bound significantly more efficiently to 4T1 cells than the non-targeted particles did. Namely, AuNR/Ag/Dox-EpCAM bound 12.5% (±0.5%) of 4T1 cells, whereas untargeted AuNR particles bound 4% (±0.1%) and AuNR/Ag/Dox bound 7.3% (±0.5%) of ﻿4T1 cells (Fig. 2d).

### AuNR/Ags-EpCAM are preferentially cytotoxic to EpCAM-expressing TNBC cells

**Fig. 3:**
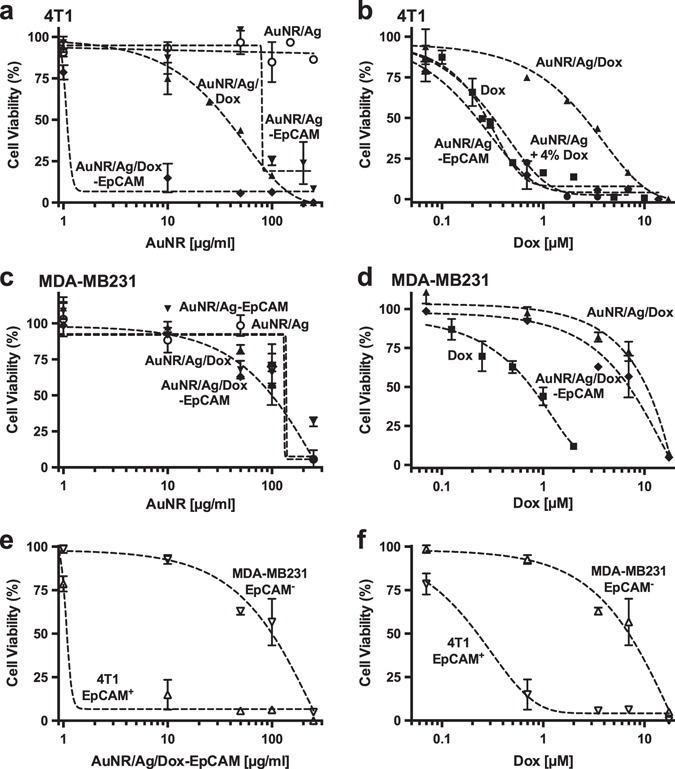
Cell viability inhibition by AuNR/Ag formulations. The effects of the different nanoconstructs on the viability of 4T1 cells as a function of **a** AuNR concentration or **b** doxorubicin (Dox) concentration and on MDA-MB231 cells as function of **c** AuNR concentration or **d** Dox concentration. The effects of AuNR/Ag/Dox-EpCAM on the viability of 4T1 cells compared to MDA-MB-231 cells as a function of **e** AuNR/Ag concentration or **f** Dox concentration. Data represent mean ± SEM. Symbols in **a**–**d** represent AuNR/Ag (○), AuNR/Ag/Dox (▲), AuNR/Ag-EpCAM (▼), AuNR/Ag/Dox-EpCAM (♦), AuNR/Ag and 4% Dox (●), Dox (■), in **e-f** 4T1 cells (∆), MDA-MB231 cells (∇)

The ED_50_, at which 50% of cells are affected, of Dox alone against TNBC-expressing EpCAM was 0.3 µM (0.16 µg/ml), whereas unmodified AuNR/Ags showed an ED_50_ greater than 250 µg/ml (Fig. 3 and Table 1). The ED_50_ was unaffected and remained at 0.3 µM when the two monotherapies, AuNR/Ags and an equivalent quantity of unconjugated Dox, were incubated together. Conjugation of Dox to the AuNR/Ags resulted in an ED_50_ of 40 µg/ml, equivalent to a Dox dose of 3 µM (based on 4% w/w loading). Conjugation of anti-EpCAM antibody to the unloaded AuNRs resulted in an ED_50_ of 80 µg/ml despite the lack of any conjugated drug molecules. AuNR/Ags conjugated with both Dox and anti-EpCAM showed an ED_50_ of 3 µg/ml (0.2 µM Dox)—a 10-fold reduction in dose compared to the untargeted Dox-loaded particle and a slight reduction compared to the combined monotherapies. The anti-EpCAM antibody by itself had no effect on cell viability (Supplementary Fig. 2).

**Table 1:** Effective doses (ED_50_) causing 50% inhibition of cell viability by the various AuNR/Ag constructs

	4T1		MDA-MB-231	
	AuNR/Ag (µg/ml)	Dox (µM)	AuNR/Ag (µg/ml)	Dox (µM)
Dox	NA	0.3	NA	0.8
AuNR/Ag	> 250	NA	150	NA
AuNR/Ag-EpCAM	80	NA	175	NA
AuNR/Ag + Dox	4	0.3	ND	ND
AuNR/Ag/Dox	40	3	150	10
AuNR/Ag/Dox-EpCAM	3	0.2	110	8

The targeting capability of anti-EpCAM was further confirmed using the low EpCAM-expressing breast cancer cell line MDA-MB-231. The ED_50_ of Dox against this cell line was found to be 0.8 μM (0.4 µg/ml), and the ED_50_ of AuNR/Ags was found to be greater than 100 µg/ml, in line with the non-cytotoxic profile on 4T1 cells. Untargeted Dox-loaded particles showed an ED_50_ of 10 µM for the Dox concentration of anti-EpCAM particles. Using anti-EpCAM antibodies as a targeting agent had little effect on the efficacy of the nanoconstruct against the EpCAM-negative cell line, displaying an ED_50_ of 8 µM as expressed in Dox concentration and 100 µg/ml in AuNRs concentration (Fig. 3). A similar differential was observed with untargeted, Dox-loaded AuNR/Ags against 4T1 cells.

### Raman and photoacoustic mapping to image EpCAM-targeting AuNR/Ags

**Fig. 4:**
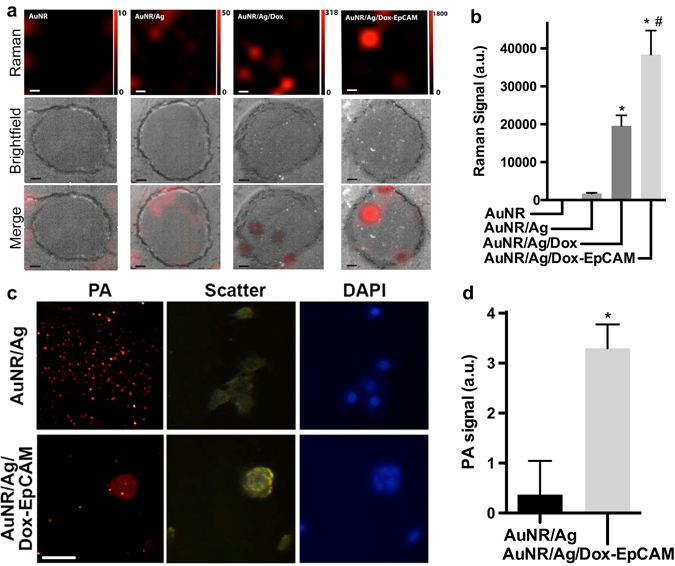
Nanoparticle visualization and quantification by SERS and photoacoustic mapping on TNBC cells. **a** Representative Raman mapping of single 4T1 cells following incubation with various AuNR constructs and **b** quantification of this signal intensity at 1080 cm^-1^. *Scale bar* in **a** represents 5 μm. **c** Representative photoacoustic images of AuNR/Ag/Dox-EpCAM particles on 4T1 cells and **d** quantification of AuNR/Ag/Dox-EpCAM photoacoustic signals on 4T1 cells. *Scale bar* ﻿in **c** represents 50 μm. ﻿Data represent mean ± SEM

SERS mapping was used to image the AuNR/Ags’ association with tumor cells. The AuNR/Ag/Dox-EpCAM nanoparticles significantly bound to the 4T1 cells compared to the untargeted constructs, AuNR/Ag and AuNR/Ag/Dox. While the untargeted AuNR/Ag emitted an integrated Raman signal of 1.8 ± 0.2 × 10^3^ a.u., the conjugation of Dox alone increased the signal strength to 20 ± 3 × 10^3^ a.u., an increase of ~11-fold. AuNR/Ag/Dox-EpCAM nanoparticles, however, displayed the greatest integrated Raman signal of 38 ± 6 × 10^3^ a.u., approximately doubling the signal compared to Dox alone (Fig. 4a, b).

Subsequently, we used PA mapping to quantify AuNR/Ag cellular uptake (Fig. 4c, d). AuNR/Ags caused a slight increase in PA signal relative to the background, but strong particle association with cells was not observed. Particles that targeted EpCAM and were loaded with Dox had a nearly 10-fold increase in cellular signal intensity (3.3 ± 0.5 a.u. for the AuNR/Ag/Dox-EpCAM particles vs. 0.37 ± 0.68 a.u. for the AuNR/Ag particles), with little to no non-cellular binding (Fig. 4c, d). We also used PA mapping to quantify AuNR binding to tumor tissue sections. Particles that targeted EpCAM had a twofold increase in signal intensity as quantified by total pixel area, 94.5 ± 6.4 a.u. for targeted particles vs. 40.1 ± 7.2 a.u. for non-targeted particles (Supplementary Fig. 3).

### In vivo distribution

**Fig. 5:**
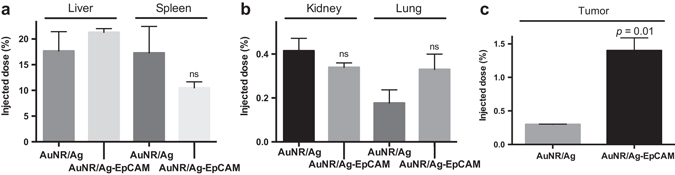
ICP-MS bio-distribution analysis of targeted nanoparticles showing a 4.5× increase in 4T1 tumors as compared to untargeted particles 24 h post injection. **a** Non-significant differences in nanoparticle accumulation in the liver and spleen between AuNR/Ag and AuNR/Ag-EpCAM particles. **b** Non-significant differences in nanoparticle accumulation in the kidney and lung between AuNR/Ag and AuNR/Ag-EpCAM particles. **c** Significant differences (*p* = 0.01) in nanoparticle accumulation in the tumor. Tumoral AuNR/Ag accumulation averaged around 0.3% of the injected dose whereas AuNR/Ag-EpCAM particles accumulated at the amount of 1.4% of the injected dose, an approximate 4.5× increase. Data represent mean ± SEM (*n* = 3 per group)

To assess the biodistribution and in vivo targeting ability of EpCAM targeting AuNRs we used ICP-MS. Based on the detected Au content we found that, compared to untargeted particles, the targeted particles significantly accumulated in TNBC tumors, on average 4.5 times more after 24 h (Fig. 5). However, no significant differences between targeted and non-targeted nanoparticle accumulation was seen in the liver or the spleen (average between 10 and 20% of the injected dose) nor the kidney and the lung (<0.5% of the injected dose).

## Discussion

Many investigators have argued EpCAM is a highly immunogenic tumor-associated antigen.^17^ It has been shown that EpCAM over-expression is required for breast cancer cell lines to proliferate, migrate, and become invasive.^12, 17^ Although this statement still holds true, the results of this study demonstrate that the amount of EpCAM expression per breast cancer cell line can differ greatly. Specifically, we showed that 4T1 TNBC cells have on average a 100-fold higher EpCAM expression than MDA-MB-231 breast cancer cells. This expression variance aligns with previous reports on protein levels^18, 19^ as well as on mRNA expression levels, where it was noted that primary and metastatic breast cancers have 100- to 1000-fold increase compared to normal breast tissue.^12^ These previous findings also correlate well with the cytotoxic profile of our AuNR/Ag/Dox-EpCAM particles: high EpCAM-expressing TNBC cells were 100-fold more sensitive than low EpCAM-expressing breast cancer cells, rendering a favorable therapeutic index.

It has been noted that EpCAM is expressed on various normal epithelia, similar to many other “tumor-associated” or “self-antigens,” which could limit its usefulness as a target.^17^ However, several EpCAM-directed antibodies are well tolerated by and seem to ignore most normal EpCAM-expressing tissues.^17^ It is now believed that EpCAM accessibility is greatly enhanced in tumor cells compared to normal epithelium, due to the overall amount of expression or the reduction in chaperone molecules, such as tetraspanin CD9, masking the EpCAM protein in the normal cell membrane.^20^ It can also be speculated that the EpCAM protein is differentially folded or glycosylated, modulating the accessibility of an antibody to certain epitopes between normal epithelial and tumor cells.

Our research groups have previously demonstrated the potent therapeutic potential of a number of gold-based and liposomal-based tumor targeting and sensitizing strategies.^9, 21–23^ The potential of multifunctional nanoparticles to allow for and improve cancer diagnostics and therapeutics simultaneously (theranostic) has motivated researchers to investigate new strategies for their use. However, the search for effective targeting strategies against specific cancer types, such as TNBC, is ongoing. For example, Das et al. described the use of nutlin-3a loaded poly (lactide-co-glycolide) nanoparticles functionalized with an EpCAM aptamer using quantum dots for imaging.^24^ Others are focusing on the diagnostic aspect, generating ultra-bright fluorescent silica nanoparticles and targeting EpCAM.^25^ In this study we present a plasmonically active, EpCAM-targeting nanodrug approach, a customized drug delivery strategy for a clinically challenging cancers. In addition, the modular makeup of our platform offers advances for therapeutically active drug treatment regimens. Theranostic anti-cancer nanoplatforms need to have a number of characteristics, ideally including: (i) relatively low bioactivity and excellent stability throughout the experimental process; (ii) low toxicity; (iii) active surfaces for tunable bio-functionalization with agent(s); (iv) strong optical absorption; and (v) ability to provide a multitude of unique spectroscopic signals (e.g., SERS, PA, fluorescent) in complex biological environments. Our platform meets all these criteria, and additionally are easily functionalized with potent anti-cancer drugs and/or targeting moieties and it provides detection and possibly quantification of cancer cells within the tumor microenvironment. The SERS signal-amplifying Ag layer (over 200 fold increase) plays a crucial role in AuNR/Ag detection, allowing for detection down to individual cells. Furthermore, these nanoparticles have strong PA signatures (several orders of magnitude more intense than other low absorbing polymeric or biological nanoparticles), which results in a complimentary approach to their detection and visualization.^26^ Recently, it was shown that SERS-based nanosystems can generate unique signals more readily detectable than regular fluorescent dyes.^27–29^ Combining SERS with the PA techniques could result in a combinatorial platform technology that can visualize and monitor these plasmonically tunable nanosystems as they deliver drugs in tumors.

There is an ongoing need for new cancer therapeutics, particularly ones that specifically target tumors. In this study, we investigated EpCAM targeting by multifunctional gold-based, silver-coated nanorods (AuNR/Ag) loaded with the conventional chemotherapeutic drug doxorubicin. This unique nanotherapeutic (AuNR/Ag/Dox-EpCAM) specifically targeted EpCAM-expressing tumors over low EpCAM-expressing tumors. Furthermore, the delivered AuNR were able to be detected by SERS and PA methods. These results represent a plasmonically active nanosystem that delivers chemotherapeutics to triple-negative breast cancer with excellent visualization and targeting capabilities, warranting further investigations for their possible use in clinical settings. This platform is well-suited for current clinically approved chemotherapeutics with dose-limiting toxicities, e.g., platinum-based or taxane-based therapies—allowing them to be administered in a targeted way and making it possible to detect them by multiple imaging techniques.

## Methods

### Synthesis of AuNRs

AuNRs were prepared using the silver ion-assisted, seed-mediated method.^30^ Briefly, the seed solution was prepared by mixing 5 ml of CTAB solution (0.2 M) with 5 ml of HAuCl_4_ (0.0005 M), then 600 μl of NaBH_4_ (0.01 M) was added with stirring for 2 min. To synthesize gold nanorods with an aspect ratio of 3, 5 ml of CTAB (0.2 M) was mixed with 150 μl of silver nitrate solution (0.004 M), then 5 ml of HAuCl_4_ (0.001 M) were added and mixed. Next, 70 µl of ascorbic acid (0.0788 M) was mixed in; finally, 12 µl of seed solution was added. The solution was kept at 30 °C for 40 min without further stirring. The resultant AuNRs were purified twice by centrifugation at 10,000 rpm for 30 min each time to remove any excess reagents.

### Synthesis of SERS-active AuNR/Ags

To enable Raman signal detection, the prepared AuNRs were covered with a 2 nm silver layer using our previously reported method.^31, 32^ Purified AuNRs were re-dispersed in 5 ml CTAB solution by sonication, then 5 ml of 1% PVP solution and 250 μl of AgNO_3_ (0.001 M) were added and gently mixed. Next, 100 μl of ascorbic acid (0.1 M) was added, then 200 μl of NaOH solution (0.1 M). The resultant silver-coated gold nanorods (AuNR/Ags) were purified twice by centrifugation and re-dispersed in deionized (DI) water. PATP was self-assembled on the surface of the nanorods to generate SERS nano-agents (AuNR/Ag/PATP): the nanorods were dispersed in an aqueous solution, then 5 μl of 10 mM PATP was added and stirred for 3 h at 45 **°**C. Unassembled PATP was removed by centrifugation at 10,000 rpm for 30 min.

### Conjugation of doxorubicin and EpCAM antibody to AuNR/Ags

AuNR/Ags were re-dispersed in 2 ml of HS-PEG-COOH (MW = 3000) at a concentration of 2 mg/ml and vigorously stirred for 15 min. Then 1.8 ml of HS-PEG (2 mg/ml in 2 mM NaCl solution) was added and kept overnight at 4 **°**C. The unbound thiolated PEG was removed by two 15-min rounds of 4000 rpm centrifugation. A two-step NHS/EDC conjugation was done to covalently link the carboxylated, PEG-covered AuNR/Ags with the corresponding antibody.^33^ Purified AuNR/Ag/PEG-COOH (4 ml) were conjugated with EpCAM antibody and 250 μg of Dox simultaneously to obtain the final conjugate AuNR/Ag/Dox-EpCAM. The antibody-tagged AuNR/Ags were washed, resuspended in 5 ml of 1× PBS solution, and kept at 4 **°**C.

### Atomic force microscopy

AuNR/Ags were imaged using AFM. The samples for AFM were prepared by dispensing the solvent (1 ×  PBS) containing the nanostructures (1000 µg/ml) on a silicon (Si) substrate at several spots. The substrate was dried overnight in a chemical fume hood. The tapping mode of Bruker Fastscan AFM (Bruker, Billerica, MA) was utilized to scan the nanostructures with a scan rate of 1 Hz and 256 samples per line. Both height and phase images were recorded during the scanning. The Bruker Nanoscope Analysis software (version 1.8) was used to refine the images.

### X-ray photoelectron spectroscopy

The nitrogen content of Dox on the AuNR/Ags was studied using XPS (K Alpha, Thermo Scientific). The data was collected at a background pressure of 1 × 10^−9^ torr, using a monochromated Al Kα *(hµ* = 1436.6 eV) X-ray source with a spot size of 400 μm in diameter. Survey scans (0–1350 eV) were taken of each sample at a pass energy (constant analyzer energy) of 200 eV and a step size of 1 eV. The collected data were referenced to the C1s’ peak to 284.5 eV based on the data obtained for adventitious carbon grown on a glass slide. Avantage software was used to analyze the results.

### Kinetics of doxorubicin release

The AuNR/Ag/Dox in a concentration of 0.2 mg/ml was re-dispersed in 2 ml of 7.4 or 5.5 buffer solutions respectively then incubated at 37 **°**C. Dox release was measured at 0.5, 1, 3, 6, and 24 h. At each time point, aliquots were centrifuged at 10,000 rpm for 30 min and the supernatant was collected, then another 2 ml of buffer solution was added. The released Dox concentration was quantified at each time point spectrophotometrically against a Dox standard curve using the 233 nm absorption peak. An acidic pH is one of the hallmarks of the tumor microenvironment, thus Dox release was assessed at physiological pH 7.4 and at a tumoral pH 5.5.^34–36^

### Cell lines

Breast cancer cells 4T1 (#CRL-2539) and MDA-MB-231 (#HTB-26) were purchased from American Type Culture Collection (ATCC) and cultured in DMEM supplemented with 10% fetal bovine serum (FBS) and 1% penicillin + streptomycin (P/S) and passaged biweekly. JAWSII cells, an immortalized dendritic cell line derived from the bone marrow of p53^−/−^ C57BL/6 mice (ATCC #CRl-11904), were grown in 10% FBS (ATCC, #30-2020) and Alpha Minimum Essential Medium (Corning, #10-022-CV), 1% P/S supplemented with 5 ng/ml murine GM-CSF (R&D Systems, 415-ML-050, Minneapolis, MN). All cell lines were cultured and maintained as previously described.^37, 38^

### Cell viability

For viability studies, cells were seeded in a 96-well plate at 1000 cells/well and allowed to adhere for at least 3 h. The cells were dosed with Dox or various nanoconstructs in a final volume of 100 µl and incubated for 72 h (5% CO_2_, 37 °C, 100% humidity). After incubation, cell viability was assessed using CCK-8 assay (Dojindo, Japan); the difference in absorbance, between 450 nm and 650 nm, was used as the metric as previously described.^39^

### Flow cytometry

Murine or human-derived cell lines (1.0 × 10^6^ cells) were washed and incubated at 4 **°**C for 30 min with the following antibodies (Affymetrix, eBiosciences): anti-mouse EpCAM (G8.8) and anti-human EpCAM (1B-7). Subsequently, cells were washed and flow cytometry was performed using an LSRFortessa (BD Biosciences, Franklin Lakes, NJ) at the Flow Cytometry Core Facility at the University of Arkansas for Medical Sciences. The data were analyzed using FlowJo software (TreeStar, Ashland, OR).

### Surface-enhanced Raman spectroscopy

For the SERS experiments cells were seeded at 10^5^ cells/well in an 8-well chamber slide (LabTek #154534). After 24 h, the media was removed and fresh complete media was added with AuNR/Ag at a concentration of 50 µg/ml. After 24 h, media was removed and the cells were washed twice with PBS, fixed with 2% paraformaldehyde (PFA) for 20 min at 4 **°**C, washed thrice with PBS and thrice with DI water, allowed to air dry, and stored at −20 °C prior to imaging.

According to previously published procedures,^40^ SERS images of the samples were collected using a confocal Raman spectrometer (Horiba Jobin Yvon LabRam HR800, Edison, New Jersey) assembled with an He–Ne laser (784 nm) and Olympus BX-51 lens with ×100 micro-objective magnitude connected to a Peltier-cooled CCD camera. The spectrometer also has a three-dimensional (*x*–*y*–*z*) automatic adjustable stage that can map Raman scanning for a specific area at a minimum distance of 1 μm. For all measurements, the Raman spectrometer was calibrated using the Si–Si Raman signal, located at a 521 per cm. The spectra were collected using 600-line/mm gratings with an 8 sec acquisition time. All data were baselined and background-corrected, then reconstructed using Prism software. Signal quantification was performed on the 1080 per  cm peak.

### Integrated photoacoustic and fluorescence flow cytometry

For photoacoustic and fluorescence flow cytometry (PAFFC), 4T1 cells were trypsinized and aliquoted at 10^5^ cells per sample. Samples were incubated on ice with 2.5 µg/ml of the various nanoconstructs and 100 µM fluorescein diacetate for 45 min, then washed twice via centrifugation, fixed with PFA (2%, 20 min, 4 °C), and washed two additional times with PBS.

The PAFFC system, as described by our group,^21^ uses a microscope platform (Nikon Eclipse E400, Nikon Instruments, Inc., Melville, NY, USA) and features an ultrasound transducer (model 6528101, 3.5 MHz, 4.5 mm in diameter; Imasonic Inc., Besançon, France) mounted over the flow cells on an XYZ positioning stage. The flow module (quartz capillary, Molex Inc., Phoenix, AZ) has a 100 μm square cross-section. Lasers were delivered and fluorescence was collected by a 20× objective (PlanFluor, Nikon Instruments, Inc.). The setup was equipped with a 820-nm diode-pumped pulsed laser (for PA detection), which had a maximal energy in the sample of 5 µJ, pulse duration of 8 ns, and pulse rate of 10 kHz (LUCE 820, Bright Solutions, Italy). Fluorescence was excited by a 488 nm laser (IQ1C45 (488-60) G26, Power Technology, Alexander, AR, USA) with 7 mW power in the sample. Laser beams formed 5 × 150 μm lines in the capillary. PA signals from the transducer were amplified (pre-amplifier 5678; bandwidth, 200 kHz–40 MHz; gain 40 dB; Panametrics NDT), digitized (PCI-5124, 12-bit, 200 MSPS, National Instruments Inc.), and recorded.^15, 26^ All data acquisition and analysis were performed using custom LabView-based software.

### Photoacoustic mapping

For PA mapping of cells, 10^5^ 4T1 cells were seeded in a 1-chamber slide (LabTek #154453) overnight. Media was then removed and fresh media was added. Next, the cells were exposed to AuNR/Ags at 25 µg/ml overnight. After incubation, the media was removed and the cells were stained with Hoechst 33342, a bisbenzimide, for 20 min. The stain was then removed, and the cells were washed twice with PBS (with 2% FBS) and fixed with 2% paraformaldehyde (20 m, 4 °C). The paraformaldehyde was removed and the cells were washed twice with PBS; a thin layer of water-soluble lubricant was added on top of the cells to reduce trailing the AuNR/Ag by the laser beam. For tissue sections, 4T1 tumors were cryopreserved and sectioned at 5 µm. These sections were fixed with acetone, rehydrated with PBS, blocked with 5% BSA, then stained with the NR constructs for 1 h. After staining, slides were washed twice with PBS; a layer of water-soluble lubricant was applied; and a chamber was affixed to the slide, sealed, and filled with H_2_O to provide acoustic transduction.

PA imaging and nanoparticle quantification was performed using a custom laser scanning PA microscope coupled to an inverted Olympus IX81 microscope (Olympus, Inc. Center Valley, PA), as described previously.^21^ Briefly, a pair of galvo mirrors (6215 H, Cambridge Technologies, Lexington, MA) steered a 532 nm laser beam across the sample in an XY raster pattern. The laser beam was focus into the sample using 10 × (UPlan, Olympus Inc.) or 2.3 × (Thorlabs, Newport, NJ) from the bottom of the sample. The pulsed excitations laser was operated at a 10 kHz pulse repetition rate. Field of view was limited by the focal area of the transducers used—120 µm and 1.2 mm for focused 20 MHz (V316, 12 mm focal distance, Olympus-NDT Inc,) and unfocused 3.5 MHz (model 6528101, 4.5 mm in diameter; Imasonic Inc., Besançon, France) transducers, respectively. The chamber slides with cells were filled with deionized water to provide acoustic coupling with a transducer. The PA signals were amplified (5662B, Panametrics) and recorded by a computer equipped with a high-speed digitizer (PCI-5124, 12-bit card, 128 MB of memory, National Instruments, Austin, TX). Control over the mirrors and system synchronization was maintained with a digital waveform generator (DG4062, Rigol, Beijun, China). Wide area imaging was performed using a 1.2 × objective and 3.5 MHz transducer in mosaic mode by shifting sample position (0.65 mm step) via a mechanical stage (Proscan II, Prior Scientific, Inc. Rockland, MA). Individual PA images were stitched together in an automated mode. Uptake of the nanoparticles by individual cells was calculated by integrating all the PA signals corresponding to cell location. Custom Image J utilized DAPI fluorescence and dark-field scattering images to identify cells in the sample and define cell boundaries. Regions of interest were manually defined and utilized to integrate raw PA signals. The background signal was calculated using control (no cells) sample. For each cell this background signal was subtracted from the integrated PA signal to account for electronic noise influence.

### Inductively coupled plasma mass spectrometry

ICP-MS was performed as recently described.^41^ In brief, Balb/c mice averaging 6–8 weeks of age (Jackson Labs, ME) were inoculated subcutaneously in the rear limb with 2 × 10^5^ 4T1 cells. After 10 days, tumors had grown to an average size of 8–10 mm in diameter at which time the animal was randomly injected intravenously via the tail vein with either 100 µl of the AuNR or AuNR/Ag/Dox-EpCAM solutions (*n* = 3 per group). Experiments were approved by the University of Arkansas for Medical Sciences Institutional Animal Care and Use Committee and performed in accordance with relevant regulations and guidelines. Tumor, liver, spleen, kidney, and lung were harvested 24 h after injection, massed, and digested at 90 °C in a solution of 0.5 ml HNO_3_ (99.999%), 0.1 ml H_2_O_2_ (30%), and 1 drop of hexane overnight. After digestion, 0.25 ml HCl (99.999%) was added, then the solution was diluted to 15 ml with 18 MΩ H_2_O and filtered using a 70 μm cell strainer (Fisher). ICP-MS was performed analyzing gold content in the tissue samples using iCAP Q (Thermo). Argon was used as a carrier gas at a flow rate of 1.05 ml/min, and a fluid flow of 0.97 ml/min. Matrix only was run between samples to reduce sample carryover.

### Statistical analysis

Unless otherwise indicated, all experiments were performed non-blinded in triplicate with at least technical duplicates in each experiment. Data are reported as mean ± SEM and were analyzed by the unpaired two-tailed *t*-test. *P* values  < 0.05 were considered statistically significant. Samples were excluded when they exceeded a deviation of 2 × SD from the means.

### Data availability statement

All data generated or analyzed during this study are included in this published article (and its Supplementary Information files).

## Electronic supplementary material

Supplementary Information

## Electronic supplementary material

**Supplementary Information** accompanies the paper on the *npj Precision Oncology* website (doi:10.1038/s41698-017-0030-1).
