# Supplementary material for: Triple-negative breast cancer targeting and killing by EpCAM-directed, plasmonically active nanodrug systems
Source: NPJ Precis Oncol. 2017 Sep 1;1:27. doi: 10.1038/s41698-017-0030-1 (PMC5871908; doi:10.1038/s41698-017-0030-1)
Supplement: Supplementary file 1 — Supplementary Information [file 41698_2017_30_MOESM1_ESM.docx]

**Supplemental Information**

**Triple negative breast cancer targeting and killing by EpCAM-directed, plasmonically active nanodrug systems**

Samir V. Jenkins,^1^ Zeid A. Nima,^2^ Kieng B. Vang,^2^ Ganesh Kannarpady,^2^ Dmitry A. Nedosekin,^3^ Vladimir P. Zharov,^3^ Robert J. Griffin,^1^ Alexandru S. Biris,^2^ and

Ruud P.M. Dings^1^

^1^Department of Radiation Oncology and the ^3^Phillips Classic Laser and Nanomedicine Laboratories, University of Arkansas for Medical Sciences, Little Rock, AR, USA

^2^Center for Integrative Nanotechnology Sciences, University of Arkansas at Little Rock, Arkansas, Little Rock AR, USA

**Corresponding Author:** Ruud P.M. Dings, 4301 W. Markham, Mail Slot #771,

Little Rock, AR 72205. Email: rpmdings@uams.edu

**Running title:** EpCAM-directed, plasmonically active nanodrug systems

**Keywords:** targeted therapy, triple negative breast cancer, nanotherapy, doxorubicin, EpCAM, photoacoustic microscopy, surface-enhanced Raman spectroscopy and therapeutic index, rational design.

**Conflict of Interest:** The authors declare no conflicts of interest.


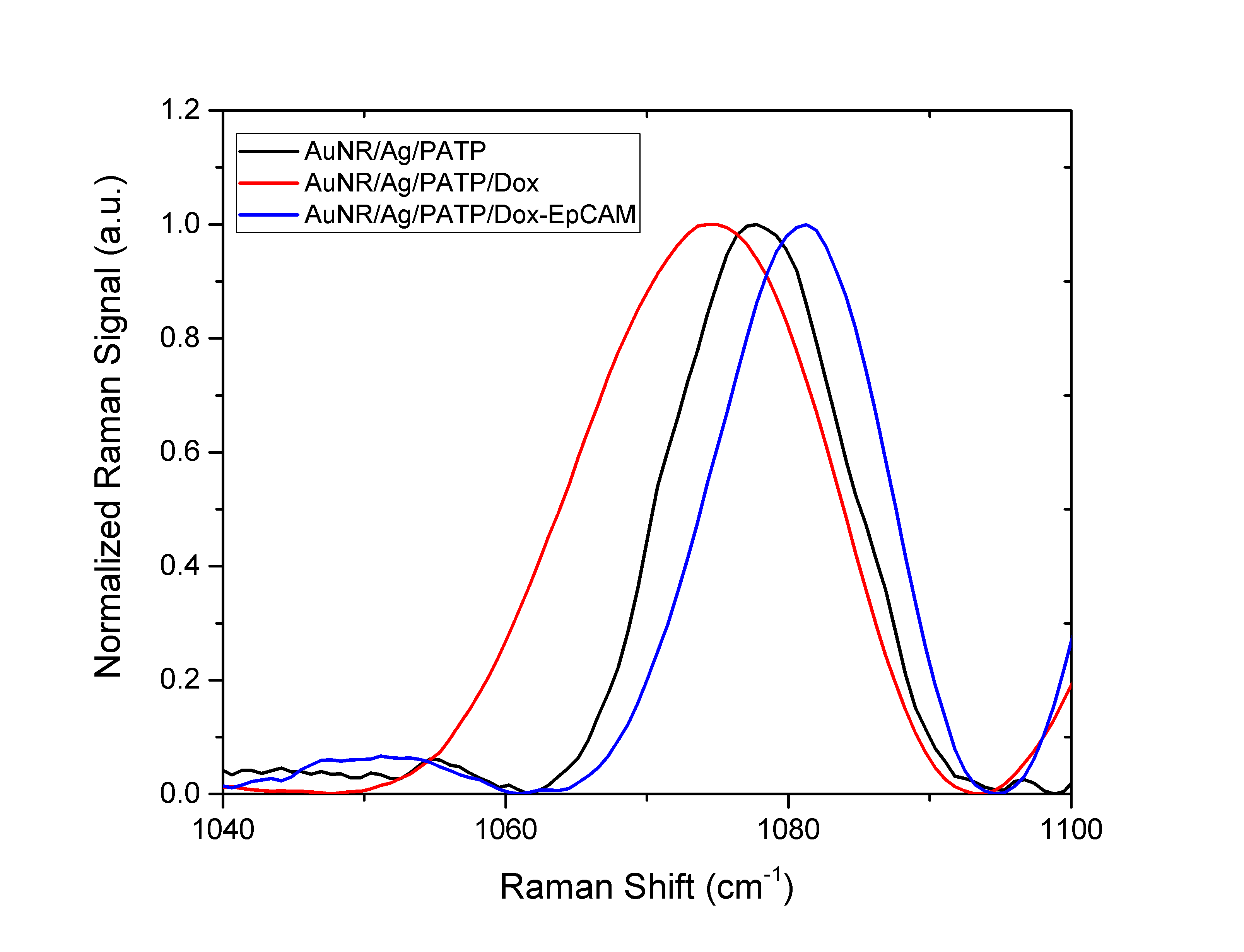


**Figure SI-1**. Raman spectra of individual particle preparations.

**Figure SI-2**. EpCAM antibody by itself does not cause cell viability inhibition.

The effects of EpCAM antibody on the viability of 4T1 cells. Please note at the ED_50_ of AuNR/Ag/Dox-EpCAM (3 µg/ml) a total of 0.05 µg/ml EpCAM Ab is present. Data represent mean +/- SD.


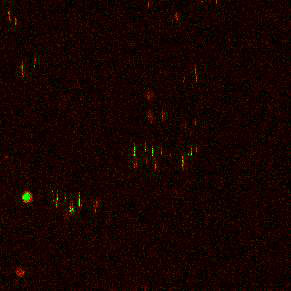

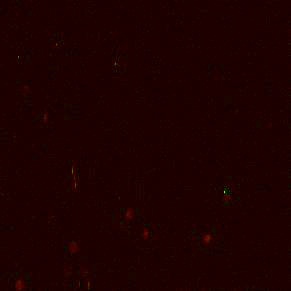


**a**

**b**

**AuNR/Ag-EpCAM**

**AuNR/Ag**

**Figure SI-3**. PA detection of targeted nanoparticles in TNBC. (**a**) Representative images of AuNR/Ag and AuNR/Ag-EpCAM particles on 4T1 tumor slections. Red = low intensity signal; Green = high intensity signal (**b**) Quantification of AuNR/Ag and AuNR/Ag-EpCAM PA signals on 4T1 tumor sections.
